# Rational approach toward COVID-19 main protease inhibitors via molecular docking, molecular dynamics simulation and free energy calculation

**DOI:** 10.1038/s41598-020-74468-0

**Published:** 2020-10-19

**Authors:** Seketoulie Keretsu, Swapnil P. Bhujbal, Seung Joo Cho

**Affiliations:** 1 Department of Biomedical Sciences, College of Medicine, Chosun University, Gwangju, 501-759 Republic of Korea; 2 Department of Cellular Molecular Medicine, College of Medicine, Chosun University, 375 Seosuk-dong, Dong-gu, Gwangju, 501-759 Republic of Korea

**Keywords:** Computational models, Virtual drug screening

## Abstract

In the rapidly evolving coronavirus disease (COVID-19) pandemic, repurposing existing drugs and evaluating commercially available inhibitors against druggable targets of the virus could be an effective strategy to accelerate the drug discovery process. The 3C-Like proteinase (3CL^pro^) of the severe acute respiratory syndrome coronavirus 2 (SARS-CoV-2) has been identified as an important drug target due to its role in viral replication. The lack of a potent 3CL^pro^ inhibitor and the availability of the X-ray crystal structure of 3CL^pro^ (PDB-ID 6LU7) motivated us to perform computational studies to identify commercially available potential inhibitors. A combination of modeling studies was performed to identify potential 3CL^pro^ inhibitors from the protease inhibitor database MEROPS (https://www.ebi.ac.uk/merops/index.shtml). Binding energy evaluation identified key residues for inhibitor design. We found 15 potential 3CL^pro^ inhibitors with higher binding affinity than that of an α-ketoamide inhibitor determined via X-ray structure. Among them, saquinavir and three other investigational drugs aclarubicin, TMC-310911, and faldaprevir could be suggested as potential 3CL^pro^ inhibitors. We recommend further experimental investigation of these compounds.

## Introduction

The coronavirus disease (COVID-19) is an acute respiratory tract disease caused by the severe acute respiratory syndrome coronavirus 2 (SARS-CoV-2) and was first reported in December 2019 in Wuhan, China^1^. The disease was declared a pandemic by the World Health Organization (WHO) on March 11, 2020^2^. Since then, it has spread to 218 countries and has infected more than 25 million people and claimed the lives of 852,000 people until September 2, 2020. The common symptoms observed in COVID-19 patients include fever, cough, fatigue, shortness of breath, and loss of smell^3^.

Epidemiological analyses have shown that the SARS-CoV-2 has a lower fatality rate (5%) but a higher transmissibility rate (2–2.5%) than those of the previously known coronaviruses Middle East respiratory syndrome (MERS) coronavirus (34.4% fatality, < 1% transmissibility) and severe acute respiratory syndrome (SARS) coronavirus (9.5% fatality, 1.7–1.9% transmissibility)^4,5^. Serial viral load analyses in COVID-19 patients using reverse transcriptase quantitative polymerase chain reaction (RT-qPCR) indicated that peak viral load was observed during the first week of symptom onset, with a median viral shedding period of 20 days^6,7^. Antibody production starts approximately 10 days after symptom onset. Cohort studies of COVID-19 patients associated old age, multiple organ dysfunction, and high blood coagulation activity on admission with increased odds of death. Sepsis, respiratory failure, acute respiratory distress syndrome (ARDS), heart failure, and septic shock were the commonly observed complications among the cohorts^7,8^. Chakraborty et al. have provided a detailed review regarding the diagnostic and proposed therapeutic options for COVID-19 treatment^9–12^.

Following the outbreak of the COVID-19 pandemic, several drug candidates from the repository of existing drugs have been tested for activity against SARS-CoV-2^13–15^. The Food and Drug Administration (FDA) has also created a special emergency program, the Coronavirus Treatment Accelerated Program (CTAP), that has reviewed 270 trials and is currently monitoring more than 570 drug development programs in the planning stage^16^. A review of the currently available literature shows that several existing antiviral drugs that target the viral replicating mechanism are under investigation for the treatment of COVID-19. The list of antiviral drugs being tested for COVID-19 includes remdesivir, hydroxychloroquine, chloroquine, lopinavir, darunavir, baloxavir, imatinib, and favipiravir^17^. Immunomodulating drugs that reduce inflammatory responses such as corticosteroids, tocilizumab, ruxolitinib, infliximab, acalabrutinib, and azithromycin are also under clinical investigation^18–21^. Various adjunctive drugs such as vitamins C and D and antithrombotics are also being considered for COVID-19 treatment^22–24^.

The ritonavir–lopinavir drug combination (Kaletra) has been used for the treatment of hospitalized patients in China and its benefits have been noted by the WHO^25^. Phase 3 clinical trials are underway to evaluate the performance and safety of the influenza drug favipiravir^26,27^. Remdesivir, an RNA-dependent RNA polymerase inhibitor, has been identified as a potential therapeutic agent for COVID-19 based on in vitro studies of SARS-CoV-2 clinical isolates^28,29^. The FDA issued an Emergency Use Authorization (EUA) for the emergency use of the drug following promising results from a placebo-controlled randomized clinical trial of remdesivir for COVID-19 treatment (https://www.fda.gov/). In a clinical study, remdesivir showed effectiveness in reducing the recovery time in COVID-19 patients. However, the drug did not contribute to significant improvement in survival rates, and the efficiency of the drug in reducing viral load in patients remained unclear. The FDA has also authorized the emergency use of the anti-malarial drugs chloroquine and hydroxychloroquine for COVID-19 treatment^30,31^. However, the clinical efficacy of these drugs remains inconclusive.

Viruses that cause diseases in humans are known to encode one or more proteases that play important roles in the viral life cycle. Proteases are ideal drug targets for viral diseases as they are responsible for cleaving the viral polyprotein, thus continuing the viral replication process^32,33^. Protease inhibitors have been used in combination drug therapy in diseases where the virus developed resistance by mutation. This strategy of using combination therapy to combat drug resistance has been successfully used in the treatment of viral diseases such as acquired immunodeficiency syndrome, in which protease inhibitors were used in combination with nucleoside reverse transcriptase inhibitors^34^.

The SARS-CoV-2 replicase enzyme encodes two polyproteins, pp1a and pp1ab, that produce all functional polypeptide units responsible for replication and transcription. Polypeptides are released by the catalytic cleavage activity of 3CL^pro^ at various subsites of the polyproteins. This cleavage process is known to be conserved in 3CL^pro^ for all coronaviruses^35,36^. Due to the important role of 3LC^pro^ in the viral replication process and the absence of a close homolog in humans, this protease has been regarded as a promising therapeutic target for COVID-19 treatment^37^. However, despite its potential, the quest for 3CL^pro^ inhibitors feasible for therapeutic use against COVID-19 has been unsuccessful so far.

Computer-aided drug discovery (CADD) methodologies have emerged as powerful tools in the drug discovery process and have been used over the last decade to identify protein inhibitors and to study protein-drug interactions and protein–protein interactions^15,38–40^. Since the development of a candidate drug into an approved drug is a long and costly process, a combination of computational methodologies such as virtual screening, docking, molecular dynamics (MD) simulation, and binding free energy evaluation, serves as a promising alternative for identifying potential drug candidates from compound libraries^41^. Cava et al. studied the mechanism of the angiotensin-converting enzyme 2 (ACE2) and its co-expressed genes using gene expression profiles in silico and suggested several interesting potential drug candidates for COVID-19^42^. Wang et al. performed virtual screening of the approved drugs and of those that are in clinical trials and identified several existing drug candidates that showed high binding affinity against 3CL^pro^^43^. Zhang et al. used in silico screening to identify potential SARS-CoV-2 inhibitors from a repository of traditional Chinese medicines^44^. Liang et al. performed MD simulation to demonstrate the binding stability of an α-ketoamide inhibitor inside the SARS-CoV-2 main protease^45^.

In this rapidly evolving pandemic, repurposing existing drugs and evaluating commercially available inhibitors against the druggable targets of SARS-CoV-2 should be an effective strategy to accelerate the drug discovery process. Consequently, taking advantage of the availability of the X-ray crystal structure of 3CL^pro^ in complex with the inhibitor N3 (PDB code 6LU7)^36^, we performed a docking-based virtual screening of the protease inhibitor database MEROPS^46^ (https://www.ebi.ac.uk/merops/) to identify potential 3CL^pro^ inhibitors.

Molecular docking and dynamic simulations were carried out to study the binding interactions of the inhibitor compounds with 3CL^pro^^47^. Binding energy calculations were performed using the molecular mechanics Poisson-Boltzmann surface area (MM-PBSA) method to evaluate the binding affinity of the compounds and to identify residues important for binding with 3CL^pro^^47,48^. The results of the modeling study were carefully analyzed to identify commercially available potential 3CL^pro^ inhibitors.

## Results

The X-ray structures of the irreversible inhibitor N3^36^ and the α-ketoamide inhibitor 13b^49^ in complex with 3CL^pro^ were retrieved from the Research Collaboratory for Structural Bioinformatics (RCSB) database. In the cell-based study by Jin et al.^36^, N3 showed inhibitory activity against SARS-CoV-2 with a half-maximal effective concentration (EC_50_) value of 16.77 μM. However, N3 covalently binds to 3CL^pro^ as an irreversible inhibitor, and its half-maximal inhibitory concentration (IC_50_) value could not be determined. The α-ketoamide inhibitor 13b showed an IC_50_ value of 0.67 μM for purified recombinant SARS-CoV-2 main protease 3CL^pro^ and also showed inhibitory activity against COVID-19 with an EC_50_ value of 4 to 5 μM in human Calu-3 cells infected with SARS-CoV-2^49^. The moderate inhibitory activity values of the existing inhibitors necessitate the development of high-affinity 3CL^pro^ inhibitors.

**Figure 1:**
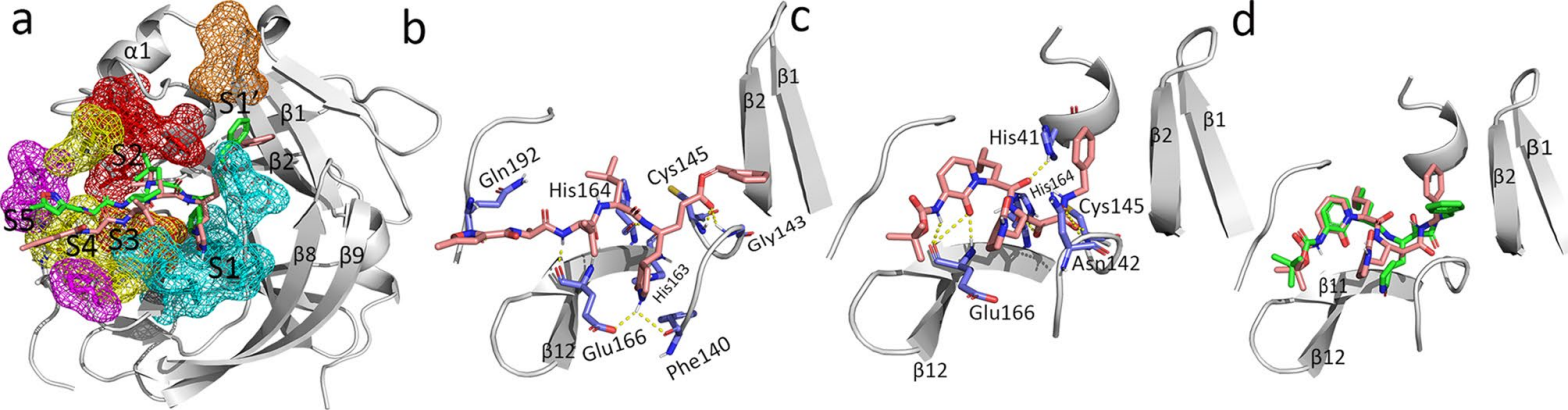
H-bond interactions of the inhibitors N3 and 13b with 3CL^pro^ from the MD simulation studies. H-bond interactions are represented by yellow dotted lines and residues forming H-bonds are shown in purple color. (**a**) The overlap between the crystal ligand pose (green) and the MD binding pose (salmon) of N3 at the binding site. The subsites S1 (cyan), S2 (red), S3 (yellow), S4 (magenta), and S1′ (salmon) are shown in mesh representation. (**b**) Binding interactions between N3 and 3CL^pro^. (**c**) Binding interactions between 13b and 3CL^pro^. (**d**) The overlap between the crystal ligand pose (green) and the MD binding pose (salmon) of 13b at the binding site of 3CL^pro^.

The 3CL^pro^ protease consists of three domains: domain 1 (residues 3–99), domain 2 (residues 100–182), and domain 3 (residues 199–307)**,** as shown in Fig. S1 (Supplementary Information)^49^. Domains 1 and 2 comprise six-stranded antiparallel β-barrels with the substrate binding site at the intersection of the two domains. As shown in Fig. 1(a), the binding site is made up of subsites S1, S2, S3, S4, and S1′, which are represented based on the binding position of the substrate polyprotein^36^. Domains 2 and 3 are connected by a hinge region (residues 182–198), which contributes to the formation of the S3 and S4 subsites. Domain 3 consists of five α-helices arranged in a globular cluster and regulates the dimerization of 3CL^pro^. The tight dimerization of 3CL^pro^ is necessary for its catalytic activity, as it leads to crucial conformational changes at the S1 subsite and subsequent binding of the substrate.

### Virtual screening

The protease inhibitor dataset consisting of 2700 compounds was retrieved from the MEROPS database^46^. Using an in-house script, we retrieved the PubChem IDs and simplified molecular-input line-entry system (SMILES) structures of the compounds from the PubChem website. 3D structures were generated using the concord module in Sybyl-X 2.1^50^ and the Open Babel package^51^. Virtual screening of the protease inhibitor dataset was performed with the Surflex-Dock^52^ program in Sybyl-X 2.1 and autodock vina. The Surflex-Dock program in Sybyl-X 2.1 uses a scoring function that includes hydrophobic, polar, repulsive, entropic, and solvation energy terms, whereas the autodock vina uses a scoring function based on steric, hydrophobic, and hydrogen bonding energy terms^52,53^. The protein file was prepared by stripping the water molecules and other heteroatoms present in it and then converting the file to pdbqt file format. The methods and parameters used for virtual screening were validated by redocking the crystal ligands N3 (PDB ID 6LU7) and 13b (PDB ID 6Y2F) into the receptor.

The total binding score from Surflex-Dock and the binding energy from autodock vina were collected and used to rank the compounds. The PubChem IDs of the 100 compounds that showed high total binding scores (Surflex-Dock) and binding energies (autodock vina) are shown in Supplementary Information Table S1. Based on the total score and binding energy, 32 compounds were selected and further studied using molecular docking, MD simulation, and free energy calculation methods. The Surflex-Dock binding scores and the autodock vina binding energies for the 32 compounds, including the reference compounds N3 and 13b, are presented in Table 1.

**Table 1:** The PubChem IDs, total scores (Surflex-Dock), autodock vina and autodock binding energies (kcal/mol), and MM-PBSA based binding energies (kJ/mol) of compounds N3, 13b, and the 32 selected compounds.

Sl. no.	PubChem ID	Total Score (Surflex Dock)	Binding energies (kcal/mol)		MM-PBSA Score (kJ/mol)
			Autodock Vina	Autodock	
1	53361968	7.1	− 8.5	− 11.0	− 151
2	451415	7.0	− 8.7	− 8.6	− 150
3	134815261	7.1	− 8.6	− 9.7	− 133
4	15942730	10.9	− 8.3	− 8.8	− 129
5	644196	10.1	− 7.1	− 7.7	− 129
6	441243	8.6	− 9.1	− 9.4	− 125
7	46178275	9.2	− 8.1	− 9.5	− 123
8	9828551	7.1	− 8.3	− 10.2	− 120
9	446837	7.9	− 8.8	− 10.8	− 115
10	132531950	9.0	− 7.8	− 9.3	− 114
11	102285029	10.3	− 7.8	− 8.8	− 111
12	11962092	8.3	− 8.7	− 9.5	− 108
13	446918	7.0	− 8.5	− 8.3	− 108
14	92727	9.7	− 8.4	− 8.8	− 104
15	45358152	9.9	− 8.1	− 7.4	− 102
16	443119	9.1	− 8.1	− 6.4	− 98
17	134691740	7.0	− 8.9	− 10.2	− 97
18	5492607	10.5	− 8.5	− 8.0	− 96
19	121304016	6.3	− 7.9	− 7.9	− 94
20	447216	7.1	− 8.7	− 9.4	− 92
21	103535	10.5	− 8.3	− 9.2	− 90
22	6918046	10.7	− 8.1	− 7.4	− 90
23	6324659	9.3	− 8	− 8.7	− 88
24	213039	7.5	− 8	− 9.1	− 81
25	5464035	9.3	− 8.2	− 9.6	− 79
26	132585244	7.2	− 8.6	− 10.7	− 77
27	134823859	7.0	− 8.7	− 10.0	− 73
28	102207029	7.0	− 8.9	− 9.8	− 63
29	21881944	7.0	− 8.6	− 8.1	3
30	4322	9.9	− 8.3	− 8.7	12
31	100997107	8.4	− 8.4	− 8.5	24
32	3451	7.0	− 8.3	− 9.8	4
**Inhibitors from crystal structures**					
33	N3 (6LU7)	10	− 7.8	− 6.3	− 150
34	13b (6Y2F)	7.8	− 9.7	− 9.7	− 99

### Molecular docking

Molecular docking of the selected 32 compounds was performed to study the binding interactions and to provide initial coordinates of the protein–ligand complexes for subsequent MD simulation studies. The X-ray crystal structure of 3CL^pro^ (PDB ID 6LU7) provided by Jin et al.^36^ was used as the receptor for this study. The docking protocol was validated by redocking the crystal ligands N3 (6LU7) and 13b (6Y2F) into the receptor. The docking showed that N3 formed H-bond interactions with residues His41, Asn142, Glu166, and Gln189 of 3CL^pro^. Compound 13b showed interactions with Asn142, Gly143, Ser144, His163, and Glu166 with the binding site residues of 3CL^pro^. The binding interactions of the inhibitors with 3CL^pro^ are shown in Supplementary Information Fig. S2. Both the X-ray structure and docked structure overlapped within a similar space inside the receptor. The docked pose of N3 overlapped with the pose in the X-ray crystal structure (PDB 6LU7) at a root mean square deviation (RMSD) value of 2.6 Å, whereas the docked pose of 13b and X-ray structure (PDB 6Y2F) showed an RMSD value of 1.8 Å. The overlaps between the autodock docked pose and the X-ray structure for both compounds N3 and 13b are shown in Fig. S5 (Supplementary Information).

The docking protocol used in docking the 3CL^pro^ inhibitors was used to dock the selected 32 compounds. The resultant binding energy values of the 32 compounds are presented in Table 1. Binding conformations of the compounds were carefully selected based on the binding energy values and also based on important non-bonded interactions observed with 3CL^pro^. The protein–ligand complexes from the docking study were used as initial coordinates in the MD simulations.

### Molecular dynamics simulation

GROMACS 2019^54^ was used to perform classical MD simulations of the selected 32 protein–ligand complexes to study the dynamic binding interactions of the compounds with 3CL^pro^.

**Figure 2:**
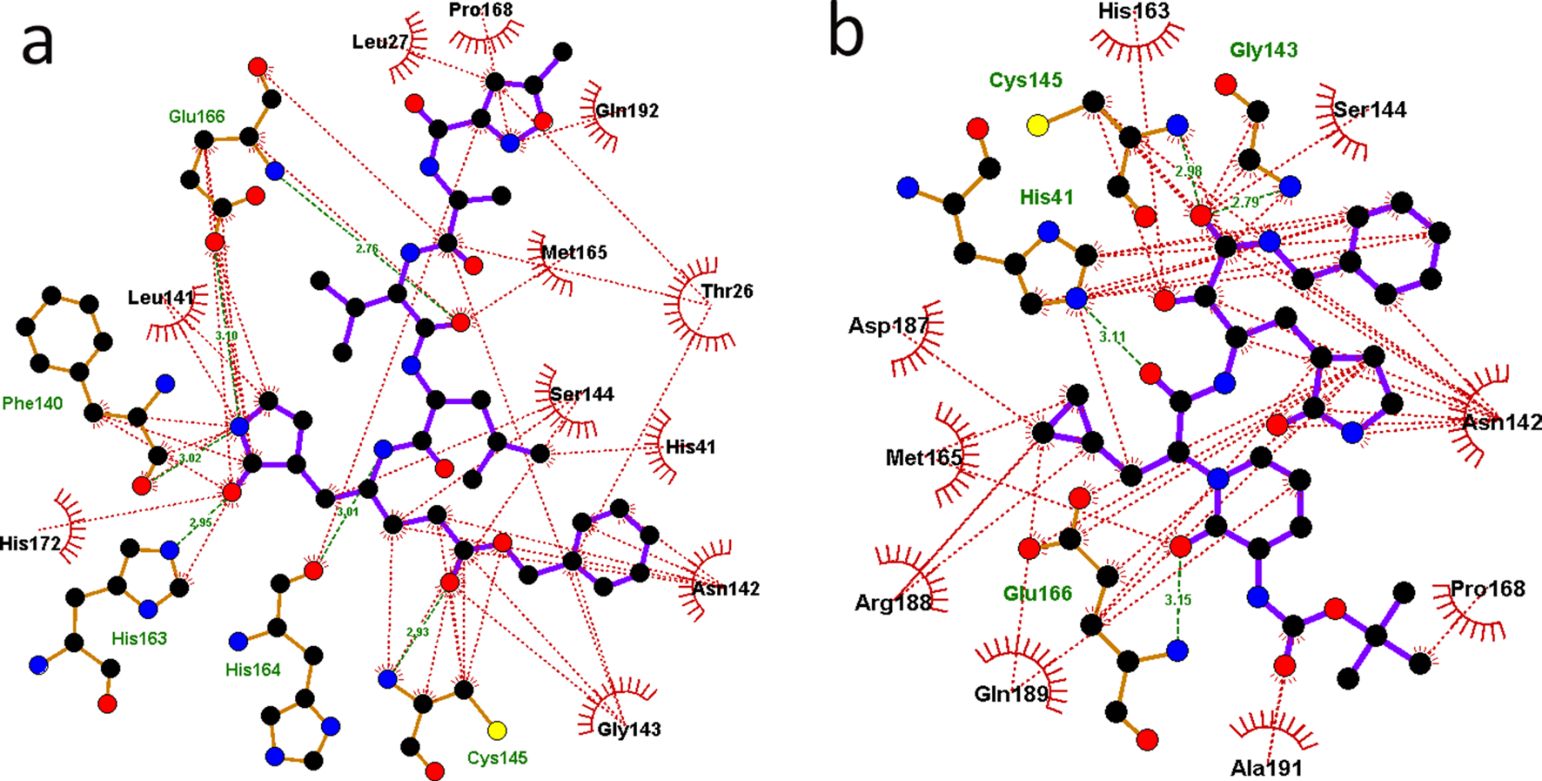
Hydrophobic interactions of the inhibitors N3 and 13b with 3CL^pro^. Hydrophobic interactions are represented by red dotted lines. Residues showing hydrophobic and H-bond interactions are written in green and black ink, respectively. Carbon, oxygen, nitrogen, and sulfur atoms are shown in black, red, blue, and yellow colors, respectively. (**a**) N3-3CL^pro^ complex. (**b**) 13b-3CL^pro^ complex.

For a comparative study, we also performed MD simulations of the N3-3CL^pro^ complex (PDB ID 6LU7) and the 13b-3CL^pro^ complex (PDB ID 6Y2F). The observed H-bond interactions and hydrophobic interactions are shown in Figs. 1 and 2, respectively. The X-ray structure of N3-3CL^pro^ showed H-bond interactions with Phe140, Gly143, His160, Glu166, Glu189, and Thr190^36^. The ligand N3 also formed a covalent bond with Cys145. However, this covalent bond with Cys145 was not observed in the MD simulation result because the standard force field (AMBER99SB) cannot account for the formation of covalent bonds. The inhibitor N3 formed H-bond interactions with Phe140, Gly143, Cys145, His163, and His164 at the S1 subsite of 3CL^pro^. H-bond interactions were also observed between N3 and the hinge residue Glu192 near the S4 subsite. Isopropyl moieties of N3 were seen at the adjacent hydrophobic subsites S2 and S3. The benzene moiety of N3 was observed near the S1′ subsite**,** as seen in the crystal structure (6LU7). The binding interactions of N3 with 3CL^pro^ are shown in Fig. 1(b). Similar binding patterns were also observed in the α-ketoamide compound 13b, where the moiety between the benzene ring and the pyridone ring formed H-bond interactions with His41, Asn142, Cys145, and His164 at the S1 subsite. The benzene ring of 13b also formed hydrophobic interactions with residues at the S1′ subsite. The pyridone ring formed H-bond interactions with Glu166 of β11 and the cyclopropyl moiety extended into the small hydrophobic subsite S2. The tert-butyloxycarbonyl protecting (Boc) group of 13b did not fully extend into the S4 subsite to form interactions with the hinge residues, as observed in the N3-3CL^pro^ complex, and instead formed hydrophobic interactions with Leu167 and Pro168 at β11. The binding interactions observed in the MD simulation of 13b-3CL^pro^ are shown in Fig. 1(c). The least-square fit RMSD of the ligands N3 and α-ketoamide 13b during the 50 ns simulations are shown in Fig. 5(a) and (b). When compared with the respective crystal ligand poses, compounds N3 (Fig. 1a) and 13b (Fig. 1d) showed RMSD values of 1.5 Å and 2 Å, respectively. Similarly, the dynamic binding interactions of the 32 compounds with 3CL^pro^ were also studied. The trajectories from the MD study were used to evaluate the free energy of binding for the selected 32 compounds. The hydrophobic interactions observed from the MD simulations of N3 and 13b are shown in Fig. 2.

### Calculation of binding free energy

**Figure 3:**
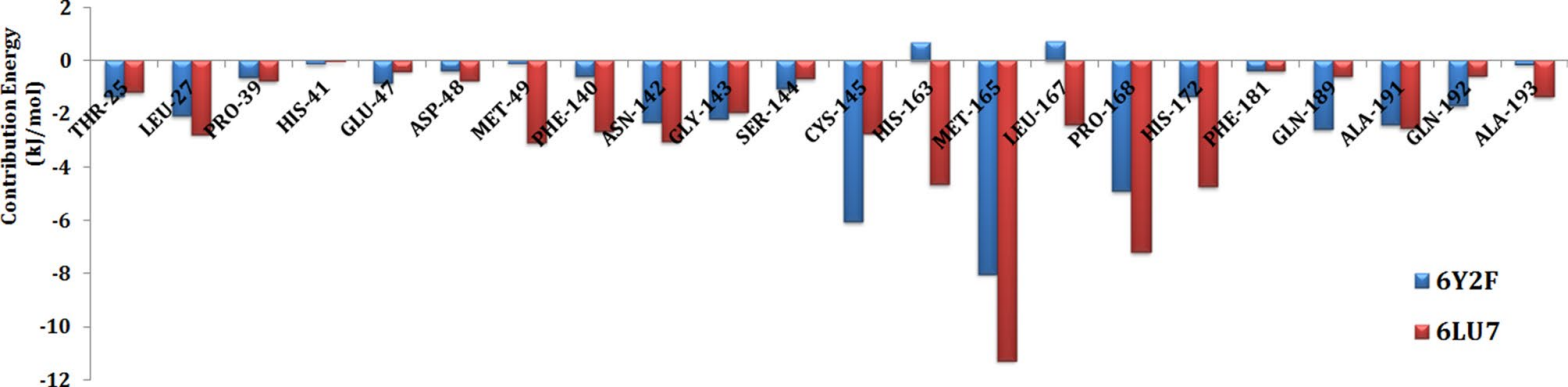
Residues with a high contribution to the total binding energy during the MD simulation of N3-3CL^pro^ (6LU7) and 13b-3CL^pro^ (6Y2F) complexes. The residues from the N3-3CL^pro^ complex and 13b-3CL^pro^ complex simulations are shown in red and blue colors, respectively.

MM-PBSA based binding energy (BE) calculations were performed for the selected 32 protein–ligand complexes, followed by evaluation of the energy contribution of the individual residues. For a comparative study, we also calculated the BE and the BE distribution for both, N3-3CL^pro^ and 13b-3CL^pro^ complexes. Compounds N3 and 13b showed BE values of − 150 kJ/mol and − 99 kJ/mol, respectively. Calculation of the BE distribution identified residues that contributed highly to the total BE, as shown in Fig. 3. The binding site residue Met165 from the S2 subsite showed the highest BE contribution, that may be attributed to the hydrophobic interaction observed with the compounds N3 and 13b. Pro168 at β11 also showed a high BE contribution, that may be attributed to the hydrophobic interaction with the methylisoxazole of N3 and the Boc group of 13b.

The residues from the S1 subsite, namely Phe140, Asn142, Gly143, Ser144, and Cys145, and the residues from β11**,** namely His163, His164, Met165, Leu167, and Pro168, were involved in both H-bond interaction and hydrophobic interactions in N3-3CL^pro^ as well as 13b-3CL^pro^ complexes. Consequently, these residues showed relatively high BE contributions (Fig. 3). Trajectory analyses also showed that the binding interactions with S1, S2, and β11 were stable throughout the simulations. However, interactions at the S1′ and S4 subsites were transient, resulting in flexible movement as indicated by the flipping of the benzene ring at the S1′ subsite and the movement of Boc and methylisoxazole at the S4 subsite, as shown in Fig. S4 (Supplementary Information).

Following the calculation of the BE for the 32 compounds, 16 compounds showed total BE values higher than the BE of compound 13b (− 99 kJ/mol), suggesting potential inhibitory activity for 3CL^pro^. Compounds 53361968 (-151 kJ/mol) and 451415 (-150 kJ/mol) showed higher BE values than the potent inhibitor N3. We also observed that 12 compounds showed BE values in the range of − 98 kJ/mol and − 63 kJ/mol, suggesting a moderate binding affinity with 3CL^pro^. Compounds 21881944, 4322, 100997107, and 3451 showed positive BE values, possibly due to non-converging simulations. The total BE values of the compounds are presented in Table 1. Based on the MM-PBSA based BE evaluations, the residue energy contributions of 10 protein–ligand complexes with high binding affinity were analyzed, as shown in Table 2. Analysis of the energy decomposition results for the selected 10 compounds showed that residues Thr25, Leu27, His41, Asp48, Met49, Leu50, Leu141, Cys145, His164, Met167, Pro168, Asp187, Gln189, and Ala191 play important roles in the binding of the compounds with 3CL^pro^. The interactions with these residues were dominated by electrostatic and hydrophobic interactions (Table 3).

**Table 2:** Residues with a high contribution to the total binding energy during the MD simulations of the complexes 441243-3CL^pro^ 451415-3CL^pro^ 446837-3CL^pro^ 53361968-3CL^pro^ 46178275-3CL^pro^ 9828551-3CL^pro^ 644196-3CL^pro^ 134815261-3CL^pro^ 15942730-3CL^pro^ and 132531950-3CL^pro^. The energy values of the residues are in kJ/mol.

Residues	441243	451415	446837	134815261	53361968	15942730	46178275	9828551	132531950	644196
THR25	− 1.16	− 4.25	− 0.89	− 2.02	− 1.56	− 2.07	− 3.03	− 0.16	− 1.94	− 0.38
Leu27	− 2.43	− 3.88	− 0.55	− 0.93	− 2.43	− 1.05	− 2.41	− 1.79	− 3.16	− 3.69
His41	− 1.31	0.11	− 4.13	− 0.36	− 8.03	− 4.21	1.78	− 0.79	− 4.73	− 1.39
Cys44	− 0.23	− 0.04	0.39	0.22	− 1.35	2.46	− 0.08	− 0.10	− 0.31	− 0.20
Asp48	− 0.28	− 0.92	− 0.56	− 2.22	− 1.63	− 1.45	− 2.28	− 1.81	− 1.68	− 0.97
Met49	− 5.81	− 6.99	− 5.29	− 6.89	− 6.68	− 6.14	− 5.97	− 2.97	− 7.65	− 2.37
Leu50	− 0.51	− 0.49	− 0.63	− 0.43	− 1.24	− 0.69	− 0.40	− 0.29	− 0.75	− 0.12
Leu141	− 2.01	− 0.67	− 0.49	− 0.52	− 1.76	− 0.36	− 3.69	− 2.67	− 0.30	− 2.68
Cys145	− 6.26	− 3.84	− 1.70	− 2.14	− 4.28	− 4.19	− 1.78	− 2.51	− 4.35	− 6.67
His164	− 5.05	− 2.89	0.57	4.14	− 0.62	1.41	− 0.27	− 1.25	2.90	− 5.98
Met165	− 9.01	− 11.29	− 9.62	− 12.06	− 10.39	− 11.13	− 2.59	− 6.64	− 10.07	− 11.23
Leu167	− 2.28	− 3.61	− 3.40	− 4.74	− 1.49	− 2.89	− 0.61	− 0.85	− 2.00	− 0.56
Pro168	− 1.54	− 2.88	− 5.76	− 4.07	− 3.42	− 7.68	− 0.33	− 0.36	− 1.11	− 1.04
Asp187	0.02	− 2.55	− 0.41	0.09	− 2.61	− 2.03	− 2.04	− 2.37	− 3.70	− 2.14
Gln189	− 7.24	− 0.09	− 3.47	− 2.68	− 6.20	− 3.55	− 3.45	− 1.92	− 8.39	1.01
Thr190	2.46	− 0.28	2.41	0.31	− 3.98	− 2.89	− 1.10	− 1.74	2.29	0.27
Ala191	− 0.98	− 0.81	− 0.95	− 1.12	− 3.09	− 2.07	0.00	− 3.92	− 1.04	− 2.02

**Table 3:** Energy contributions of the various energetic terms to the total binding energies of the inhibitors with 3CL^pro^.

Complexes	Van der Waals (kJ/mol)	Electrostatics (kJ/mol)	Polar solvation (kJ/mol)	Non-polar (kJ/mol)	Total binding energy (kJ/mol)
441243-3CL^pro^	− 256	− 81	238	− 26	− 125
451415-3CL^pro^	− 293	− 44	217	− 29	− 150
446837-3CL^pro^	− 217	− 60	185	− 23	− 115
53361968-3CL^pro^	− 276	− 33	184	− 26	− 151
46178275-3CL^pro^	− 213	− 38	152	− 24	− 123
9828551-3CL^pro^	− 215	− 72	188	− 21	− 120
644196-3CL^pro^	− 231	− 88	214	− 24	− 129
134815261-3CL^pro^	− 262	− 54	210	− 27	− 133
15942730-3CL^pro^	− 239	− 91	225	− 24	− 129
132531950-3CL^pro^	− 254	− 53	219	− 26	− 114
N3-3CL^pro^	− 300	− 108	286	− 27	− 150
13b-3CL^pro^	− 229	− 78	232	− 23	− 99

## Discussion

**Figure 4:**
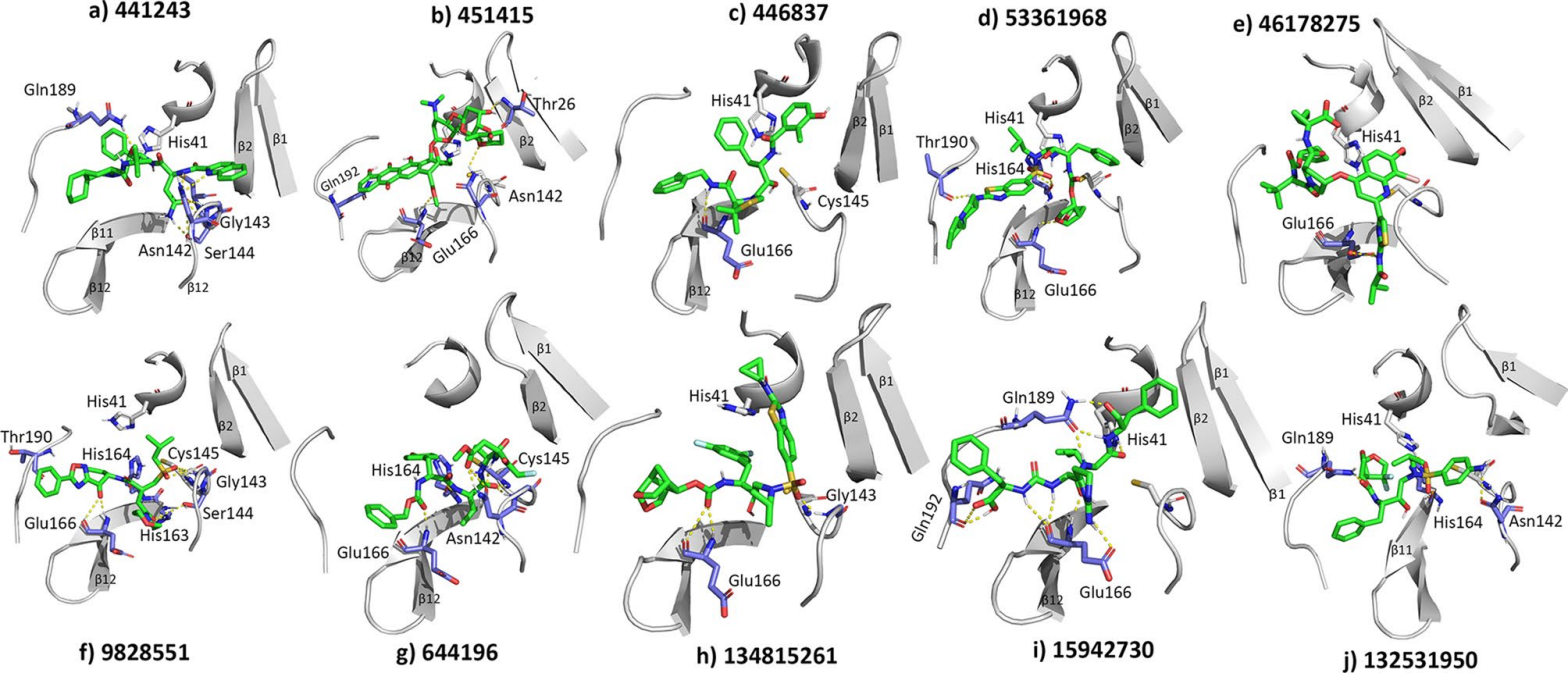
H-bond interactions of the inhibitors with 3CL^pro^. The protein and ligand are shown in gray and green colors, respectively. H-bond interactions are represented by yellow dotted lines and residues forming H-bonds are shown in purple color. (**a)** 441243-3CL^pro^ (**b**) 451415-3CL^pro^ (**c**) 446837-3CL^pro^ (**d**) 53361968-3CL^pro^ (**e**) 46178275-3CL^pro^ (**f**) 9828551-3CL^pro^ (**g**) 644196-3CL^pro^ (**h)** 134815261-3CL^pro^ (**i**) 15942730-3CL^pro^ and (**j**) 132531950-3CL^pro^.

**Figure 5:**
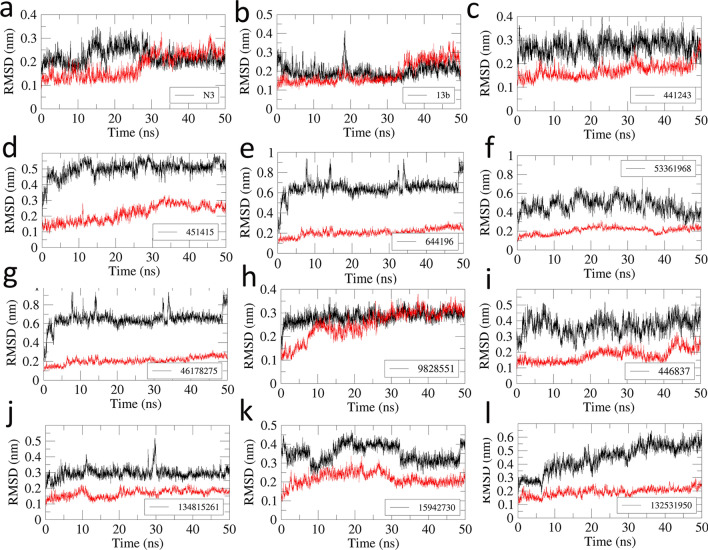
Least Square Fit Root Mean Square Deviation (RMSD) of the protein (red) and ligand (black) from the 50 ns MD simulations. (**a**) N3-3CL^pro^ (**b**) 13b-3CL^pro^ (**c**) 441243-3CL^pro^ (**d**) 451415-3CL^pro^ (**e**) 644196-3CL^pro^ (**f**) 53361968-3CL^pro^ (**g**) 46178275-3CL^pro^ (**h**) 9828551-3CL^pro^ (**i)** 446837-3CL^pro^ (**j**) 134815261-3CL^pro^ (**k**) 15942730-3CL^pro^ and (**l**) 132531950-3CL^pro^.

We selected 10 compounds that showed high potential for 3CL^pro^ inhibition based on the total binding free energy to analyze the structural features critical for binding with 3CL^pro^. The structures of the selected compounds are presented in Table 4**.** The binding interactions with 3CL^pro^ and the RMSD values for the 10 compounds are shown in Figs. 4 and 5, respectively.

**Table 4:**
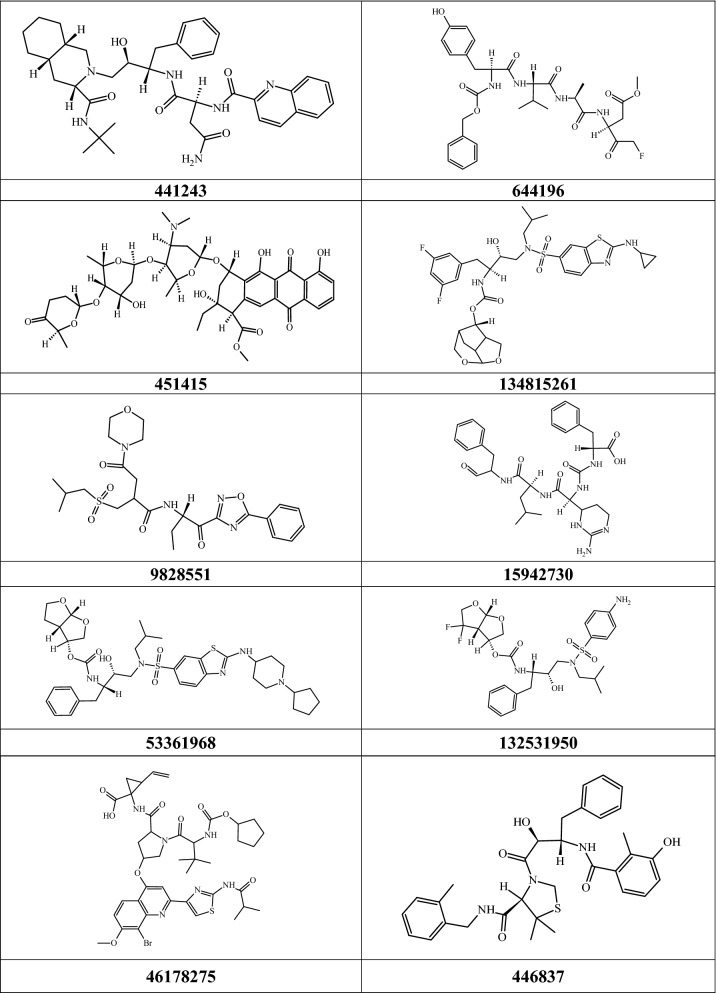
Structures of the 10 compounds selected on basis of binding energy (MM-PBSA).

Compound 441243 formed H-bond interactions with Gln189 and His41, while forming multiple interactions at the S1 subsite with Asn142, Ser144, and Gly143. Compounds 451415 (− 150 kJ/mol) and 53361968 (− 151 kJ/mol), which showed relatively high binding energy values (Table 3), had a relatively less number of H-bond interactions. However, further analysis showed that these compounds had relatively high hydrophobic energy contributions, resulting in higher total binding energy values. The donor nitrogen atoms of compound 446837 formed two H-bond interactions: with His41 and Glu166. Compound 46178275 showed only one stable H-bond interaction with Glu166. However, the oxygen and donor nitrogen atoms of 46178275 near the hinge region could form transient interactions with Gln189 and Thr190. Compound 15942730 showed the highest number of H-bonds, forming multiple interactions with His41, Glu166, Gln189, and Gln192. In the binding energy analysis of the 15942730-3CL^pro^ complex (Table 3), the contribution of the electrostatic component to the total binding energy was − 91 kJ/mol, which was higher than that of the other selected compounds. Compounds 9828551 and 644196 formed several H-bond interactions with residues from β11 and S1. Consequently, these two compounds showed high electrostatic energy terms in the binding energy calculations. Compound 134815261 formed H-bond interactions with Glu166 and Gly143. Compound 13231950 formed H-bond interactions with His41, Asn142, His164, and Gln189. The analyses suggested that compounds showing higher binding affinities with 3CL^pro^ were able to form H-bond interactions with residues from multiple subsites and also showed higher number of hydrophobic interactions.

From the analyses of the binding interactions, we observed that the interactions of the compounds with the S1 subsite residues such as His41, Asn142, Gly143, Ser144, and the Glu166 residue of β11 were crucial for stable interaction with 3CL^pro^. These interactions with the S1 and β11 residues were also observed in experimental studies^36,49^. Interactions of the compounds at the S2 subsite were predominantly hydrophobic. Since the S2 subsite is a small hydrophobic pocket, compounds with substituents such as isopropyl and cyclopropyl, which can fit into the hydrophobic pocket, could be promising 3CL^pro^ inhibitors, as in the cases of inhibitor 13b (Fig. 2b), compound 451415/aclarubicin, and 53361968/TMC-310911^55^ (Supplementary Information Fig. S3). It was also observed that compounds with substituents that extend into the S4 subsite tend to show higher binding energies. The compounds 451415/aclarubicin^56^ and 53361968, which formed H-bonds (Fig. 4b and d) and hydrophobic interactions (Supplementary Information Fig. S3) at the S4 subsite showed relatively high total BE of − 150 kJ/mol and − 151 kJ/mol, respectively, suggesting the importance of these interactions in 3CL^pro^ inhibition. This observation was also made in the experimental study of inhibitor 13b, wherein removing substituents that extended into the S4 subsite reduced the activity value^49^. Having substituents that extend into the S4 subsite induced conformational changes at the hinge region between subunits 1 and 2, as noted by Zhang et al.^49^. However, the exact mechanism behind the conformational change leading to increased affinity for 3CL^pro^ remains unclear. From interaction studies, it was observed that having substituents that form hydrophobic interactions at the S1′ subsite was important for binding with 3CL^pro^. These interactions at S1′ were dominated by hydrophobic interactions, as observed in the interactions of N3 and 13b (Fig. 2) and also in the cases of compounds 446837/KNI-764^57^ (− 115 kJ/mol), 53361968^55^ (− 150 kJ/mol), and 15942730/chemostatin^58^ (− 129 kJ/mol). However, in the case of compound 451415, that lacks an extended hydrophobic benzyl substituent, H-bond interaction was observed with Thr26 at the S1′ subsite (Fig. 4b). From these observations, we speculate that having substituents that can form hydrophobic and H-bond interactions with S1′ residues may increase the binding affinity since having both hydrophobic and H-bond interactions with 3CL^pro^ closely emulates the substrate-binding pattern^59^. Binding energy decomposition for individual residues identified His41, Met49, Met165, and Glu189 as key locations. These residues were also identified as important hotspot residues by Wang et al.^43^ in a recent study. Additionally, analysis of BE decomposition also revealed that the residues Thr25, Leu27, Asp48, Leu50, Leu141, Cys145, His164, Leu167, Pro168, Asp187, and Ala191 were significant for the binding of the inhibitors with 3CL^pro^.

The absorption, distribution, metabolism, and excretion (ADMET) properties of the compounds were also evaluated using the pkCSM server^60^ and the results are presented in Table S2 (Supplementary Information). In the ADMET analyses, compounds that showed an intestinal absorption value of less than 30% were considered to have poor absorption rates. Except for compound 15942730, all the selected compounds showed reasonable intestinal absorption rates. Steady-state volume of distribution (VDss) represents the degree to which the compounds are distributed in the body rather than the plasma, and compounds with log (VDss) values greater than − 0.15 are considered to have a reasonable distribution rate. All the compounds in Supplementary Information Table S2 except 46178275 and 15942730 showed VDss values greater than − 0.15, indicating that the compounds have satisfactory distribution rates. Analyses of the metabolism results suggest that the compounds are poor cytochrome P450 inhibitors. Compounds with positive results for the CYP3A4 substrate test suggest that they can be metabolized by cytochrome P450. The selected compounds also showed a reasonable total clearance rate from the body, except compound 446837. The negative Ames toxicity test results suggest that the compounds have poor mutagenic potential.

Being in the middle of the COVID-19 pandemic, availability of these compounds is crucial for in vivo and in vitro experimental studies. Hence, we checked the availability of the selected compounds in commercial libraries by referencing vendor data through the PubChem website and the ZINC database^61^. The details regarding the ZINC ID and the distributor (vendor) of the compounds are provided in Table 5. Compound 441243/saquinavir^62^ is an antiretroviral protease inhibitor approved by the FDA for the treatment of human immunodeficiency virus (HIV) infection. Recently, several computational studies have also reported the encouraging binding ability of saquinavir with 3CL^pro^^25,63^. The high binding affinity of saquinavir observed in our study, as well as other by independent research groups, indicates its potential as a 3CL^pro^ inhibitor. The registry of clinical trials maintained by the United States National Library of Medicine under the National Institute of Health (NIH) showed that compound 451415/aclarubicin is an anthracycline drug and has been under evaluation (phase 2 clinical trial) for combination therapy against acute myeloid leukemia (AML). Compound 53361968, which showed the highest BE value (− 151 kJ/mol), is an investigational protease inhibitor that is currently being studied for HIV-1 infection treatment^64^. Compound 46178275/faldaprevir is a hepatitis C virus protease inhibitor currently being studied for the treatment of hepatitis C^65^. Additionally, compounds 132531950 (− 114 kJ/mol), 102285029 (− 111 kJ/mol), 11962092 (− 108 kJ/mol), 446918 (− 108 kJ/mol), 92727 (− 104 kJ/mol), and 45358152 (− 102 kJ/mol) also showed BE values greater than that of the inhibitor in the X-ray structure (α-ketoamide with a BE value of − 99 kJ/mol), suggesting that these compounds may have higher inhibitory activity against 3CL^pro^.

**Table 5:** The binding energies, ZINC compound IDs, and the distributor/vendor names and vendor compound IDs of the 10 compounds selected on basis of high MM-PBSA-based binding energy evaluation.

PubChem ID	Binding energy (kJ/mol)	ZINC compound ID	Vendor	Vendor compound ID
441243 (saquinavir)	− 125	ZINC3914596	Molport	MolPort-000-883-824
451415 (aclarubicin)	− 150	ZINC8101053	Molport SC Economical	MolPort-004-845-383
446837 (KNI-764)	− 115	ZINC3941126	eMolecules	92333721
53361968 (TMC-310911)	− 151	ZINC98208561	Synblock Inc	SB17102
46178275 (faldaprevir)	− 123	ZINC150339145	Compound Cloud	42601552, 56594927
9828551	− 120	ZINC137293978	eNovation Chemicals	D676701
644196	− 129	ZINC78938888	Ambinter	Amb19930411
134815261	− 133	NA	NA	NA
15942730 (chemostatin)	− 129	ZINC3947583	NA	NA
132531950	− 114	ZINC224699399	NA	NA

*NA represents not available.

## Conclusion

In this ongoing COVID-19 pandemic, CADD methodologies can be used effectively to accelerate the process of developing therapeutic agents for the treatment of this disease. In this study, we used docking-based virtual screening to search the protease inhibitor database (MEROPS) to identify potential inhibitors of the SARS-CoV-2 main protease 3CL^pro^. Molecular docking and dynamics simulations were carried out to study the binding interactions. Binding free energy calculations were performed to identify potential 3CL^pro^ inhibitors. The study identified saquinavir, which is an approved drug for HIV-1 treatment, and several other investigational drugs, such as aclarubicin, TMC-310911, and faldaprevir. We also assessed the commercial availability of the compounds, which could be useful for experimental researchers. Analysis of the binding interactions revealed that electrostatic interactions with residues from the S1 subsite and the β-strand (β11) were important for the inhibition of 3CL^pro^. Compounds possessing substituents that extend into the S4 subsite induced conformational changes at the hinge between subunit 1 and subunit 2 and showed higher binding affinity. Compounds with high binding energies showed either hydrophobic or electrostatic interactions at the S1′ subsite. These structural features may be harnessed to design potent 3CL^pro^ inhibitors. Using CADD methods, we identified 15 compounds with a binding affinity greater than that of the inhibitor inside 3CL^pro^ in the X-ray structure (α-ketoamide). We suggest further experimental investigation of these compounds.

## Methods

### Data preparation

The X-ray crystal structure of 3CL^pro^ in complex with the inhibitor N3 (PDB ID 6LU7) prepared by Jin et al. was used as the receptor for our study^36^. The heteroatoms and water molecules were removed from the protein file for further study.

A total of 2700 protease inhibitors were collected from the MEROPS database. MEROPS is a database of proteases and their inhibitors^46^. The two-dimensional (2D) structures provided in the simplified molecular-input line-entry system (SMILES) format and the PubChem IDs of the compounds were collected from the PubChem website (https://pubchem.ncbi.nlm.nih.gov/) using an in-house script. The 2D structures were converted to three-dimensional (3D) structures using the concord module in Sybyl-X 2.1^50^.

### Virtual screening

Docking-based virtual screening was performed using the Surflex-Dock module^52^ in Sybyl-X 2.1 and the autodock vina^53^ program to identify potential 3CL^pro^ inhibitors. Since Surflex-Dock and autodock vina use different approaches in scoring the binding affinity of the compounds, using both methods increased the credibility of the virtual screening results.

### Surflex-Dock

The protein structure was prepared by adding hydrogen atoms and assigning Amber 7FF99 atom types, followed by brief energy minimization. During ligand preparation, a general cleanup process was carried out by filling valences and removing duplicates and compounds that are not drug-like. A computational representation of the binding site, called the protomol, was generated based on the crystal ligand coordinate, as shown in Fig. S1 (Supplementary Information). The protomol was used to direct the initial placement of the ligands during docking. Virtual screening was carried out via the Surflex-Dock program using a molecular similarity-based search engine. Binding interactions were evaluated using an empirical scoring function based on hydrophobic, polar, repulsive, entropic, and solvation energy terms.

### Autodock vina

3D coordinates of the compounds bearing partial charges were generated and saved in the pdbqt format. The receptor coordinates and grid parameters were generated using autodock tools^66^. The virtual screening process and the analysis of the results were performed using in-house scripts that incorporated the autodock vina program. The binding energies of the compounds were analyzed and used to rank the compounds.

### Molecular docking

Molecular docking was performed to evaluate the binding energy and to provide initial coordinates and topology parameters for the MD simulations. The docking procedure was validated by extracting the irreversible inhibitor N3^36^ (PDB ID 6LU7) and the α-ketoamide inhibitor 13b^49^ (PDB ID 6Y2F) from the crystal structures and docking them back into the receptor.

During the molecular docking of the compounds, the binding pose of the selected compounds from the virtual screening was used as the input. Polar atoms were added to the protein and Kollman charges were added as partial charges. A grid box with dimensions 60 × 60 × 60 centered at the coordinates X =  − 10, Y = 13, and Z = 70 was used to represent the search area. The Lamarckian genetic algorithm (LGA) was used to perform the docking process, generating 100 conformations for each compound. Based on the binding energy and binding interactions with the receptor, a representative binding pose for the ligands was selected.

### Molecular dynamics simulation

Classical MD simulations were carried out on selected compounds, using GROMACS 2019^54^, to evaluate their binding interactions with 3CL^pro^. The protein–ligand complexes from the docking study were used for the MD simulation study. The ligands were parameterized with the general amber force field (GAFF)^67^ using the Acpype program^68^. Protein topology and coordinate files were generated using the Amber99SB force field provided in GROMACS. The protein–ligand complex was contained in a dodecahedron and solvated with TIP3P water. Counter ions were added to neutralize the solvated system followed by quick energy minimization with the steepest descent minimization algorithm. This was followed by a restrained constant number of particles, volume, and temperature (NVT) ensemble equilibration for 500 ps and a constant number of particles, pressure, and temperature (NPT) ensemble for 1 ns equilibration. Thermodynamic properties such as pressure, density, potential energy, and temperature of the systems were monitored to ensure adequate equilibration before the production run. The particle mesh Ewald method was used to calculate the long-range electrostatics. Modified Berendsen thermostat and Parrinello-Rahman barostat were used for temperature and pressure coupling, respectively. Finally, unrestrained 50 ns production simulations were carried out for the systems at 310 K and 1 bar atmospheric pressure. The MD simulation procedure used here has been used in several protein–ligand interaction studies by our group and others^39,40^.

### Calculation of binding free energy

The g_mmpbsa package developed by Kumari et al. was used to calculate the binding free energy or simply, the binding energy (BE) of the protein–ligand complexes. The g_mmpbsa program used subroutines sourced from GROMACS and APBS packages to integrate high-throughput molecular dynamics simulation with binding energy calculations^48^.

The vacuum potential energy was calculated from the bonded and non-bonded interactions based on the molecular mechanics (MM) force field. The electrostatic and van der Waals (E_vdw_) energy contributions were calculated based on Coulomb potential and Lennard–Jones potential functions, respectively. During the evaluation of the free energy of solvation, the polar contribution was calculated by solving the Poisson-Boltzmann equation. The non-polar contribution was calculated based on the assumption that the non-electrostatic solvation energy is linearly related to the solvent-accessible surface area (SASA). The non-polar energy term (G_nonpolar_) includes both repulsive and attractive forces between the solute and solvent developed due to cavity (G_cavity_) formation as well as the van der Waals interaction (G_vdW_). This can be represented by the equation below^40,69^.

$${\mathrm{G}}_{\mathrm{nonpolar}}={\mathrm{G}}_{\mathrm{cavity}}+{\mathrm{G}}_{\mathrm{vdW}}$$

During the calculation of the BE, snapshots were generated from the equilibrated region of the MD trajectory. Energy components were evaluated for 51 snapshots extracted every 0.1 ns from the trajectory. The decomposition of the energy term to individual residues was carried out using the MmPbsaDecomp.py script provided with the g-mmpbsa package. The default parameters set by Kumari et al. were used for all the calculations.

The Figs. 1, 4, S1, S2 and S4 were rendered using the pymol v2.1 software (https://pymol.org/2/). The Fig. 2 was generated using the LigPlot^+^ software (https://www.ebi.ac.uk/thornton-srv/software/LigPlus/).

## Supplementary information

Supplementary Information 1

## Data Availability

All relevant data are contained within the manuscript and the supplementary material. Additional raw data will be available upon request.

## References

[1] Surveillances V (2020). The epidemiological characteristics of an outbreak of 2019 novel coronavirus diseases (COVID-19)—China, 2020. China CDC Wkly..

[2] Cucinotta D, Vanelli M (2020). WHO declares COVID-19 a pandemic. Acta Bio-Med.: Atenei Parmensis.

[3] Chen Y, Li L (2020). SARS-CoV-2: virus dynamics and host response. Lancet Infect. Dis..

[4] Mahase E (2020). Coronavirus: covid-19 has killed more people than SARS and MERS combined, despite lower case fatality rate. Br. Med. J. Publish. Group.

[5] Li LQ (2020). COVID-19 patients' clinical characteristics, discharge rate, and fatality rate of meta-analysis. J. Med. Virol..

[6] Pan Y, Zhang D, Yang P, Poon LL, Wang Q (2020). Viral load of SARS-CoV-2 in clinical samples. Lancet Infect. Dis..

[7] Zhou F (2020). Clinical course and risk factors for mortality of adult inpatients with COVID-19 in Wuhan, China: A retrospective cohort study. Lancet.

[8] To KK-W (2020). Temporal profiles of viral load in posterior oropharyngeal saliva samples and serum antibody responses during infection by SARS-CoV-2: An observational cohort study. Lancet Infect. Dis..

[9] Chakraborty C, Sharma A, Sharma G, Bhattacharya M, Lee S (2020). SARS-CoV-2 causing pneumonia-associated respiratory disorder (COVID-19): Diagnostic and proposed therapeutic options. Eur. Rev. Med. Pharmacol. Sci..

[10] Saha A (2020). Probable molecular mechanism of remdesivir for the treatment of COVID-19: Need to know more. Arch. Med. Res..

[11] Chakraborty C (2020). Consider TLR5 for new therapeutic development against COVID-19. J. Med. Virol..

[12] Chakraborty C (2020). Extensive partnership, collaboration, and teamwork is required to stop the COVID-19 outbreak. Arch. Med. Res..

[13] Touret F (2020). In vitro screening of a FDA approved chemical library reveals potential inhibitors of SARS-CoV-2 replication. Sci. Rep..

[14] Jácome R, Campillo-Balderas JA, de León SP, Becerra A, Lazcano A (2020). Sofosbuvir as a potential alternative to treat the SARS-CoV-2 epidemic. Sci. Rep..

[15] Trezza A, Iovinelli D, Prischi F, Santucci A, Spiga O (2020). An integrated drug repurposing strategy for the rapid identification of potential SARS-CoV-2 viral inhibitors. Sci. Rep..

[16] D’Acquarica I, Agranat I (2020). Chiral switches of chloroquine and hydroxychloroquine: Potential drugs to treat COVID-19. Drug Discov. Today..

[17] Javorac D (2020). An overview of safety assessment of the medicines currently used in the treatment of COVID-19 disease. Food Chem. Toxicol..

[18] Perez-Moreiras JV (2018). Efficacy of tocilizumab in patients with moderate-to-severe corticosteroid-resistant Graves orbitopathy: A randomized clinical trial. Am. J. Ophthalmol..

[19] Jagasia M (2020). Ruxolitinib for the treatment of steroid-refractory acute GVHD (REACH1): A multicenter, open-label phase 2 trial. *Blood*. Am. J. Hematol..

[20] Byrd JC (2020). Acalabrutinib monotherapy in patients with relapsed/refractory chronic lymphocytic leukemia: Updated phase 2 results. *Blood*. Am. J. Hematol..

[21] Saha A (2020). Tocilizumab: A therapeutic option for the treatment of cytokine storm syndrome in COVID-19. Arch. Med. Res..

[22] Carr AC (2020). A new clinical trial to test high-dose vitamin C in patients with COVID-19. Crit. Care.

[23] Grant WB (2020). Evidence that vitamin D supplementation could reduce risk of influenza and COVID-19 infections and deaths. Nutrients.

[24] Bikdeli B (2020). COVID-19 and thrombotic or thromboembolic disease: Implications for prevention, antithrombotic therapy, and follow-up: JACC state-of-the-art review. J. Am. Coll. Cardiol..

[25] Harrison C (2020). Coronavirus puts drug repurposing on the fast track. Nat. Biotechnol..

[26] Cai Q (2020). Experimental treatment with favipiravir for COVID-19: An open-label control study. Engineering.

[27] Du YX, Chen XP (2020). Favipiravir: Pharmacokinetics and concerns about clinical trials for 2019-nCoV infection. Clin. Pharmacol. Ther..

[28] Wang M (2020). Remdesivir and chloroquine effectively inhibit the recently emerged novel coronavirus (2019-nCoV) in vitro. Cell Res..

[29] Cao Y, Deng Q, Dai S (2020). Remdesivir for severe acute respiratory syndrome coronavirus 2 causing COVID-19: An evaluation of the evidence. Travel Med. Infect. Disease.

[30] Colson P, Rolain J-M, Lagier J-C, Brouqui P, Raoult D (2020). Chloroquine and hydroxychloroquine as available weapons to fight COVID-19. Int. J Antimicrob. Agents.

[31] Jaffe S (2020). Regulators split on antimalarials for COVID-19. Lancet.

[32] Kräusslich H-G, Wimmer E (1988). Viral proteinases. Annu. Rev. Biochem..

[33] Tong L (2002). Viral proteases. Chem. Rev..

[34] Yilmaz NK, Swanstrom R, Schiffer CA (2016). Improving viral protease inhibitors to counter drug resistance. Trends Microbiol..

[35] Hilgenfeld R (2014). From SARS to MERS: Crystallographic studies on coronaviral proteases enable antiviral drug design. FEBS J..

[36] Jin Z (2020). Structure of Mpro from COVID-19 virus and discovery of its inhibitors. Nature.

[37] Anand K, Ziebuhr J, Wadhwani P, Mesters JR, Hilgenfeld R (2003). Coronavirus main proteinase (3CLpro) structure: Basis for design of anti-SARS drugs. Science.

[38] Sliwoski G, Kothiwale S, Meiler J, Lowe EW (2014). Computational methods in drug discovery. Pharmacol. Rev..

[39] Keretsu S, Bhujbal SP, Cho SJ (2019). Computational study of paroxetine-like inhibitors reveals new molecular insight to inhibit GRK2 with selectivity over ROCK1. Sci. Rep..

[40] Keretsu S, Bhujbal SP, Cho SJ (2020). Molecular modeling studies of pyrrolo [2, 3-d] pyrimidin-4-amine derivatives as JAK1 inhibitors based on 3D-QSAR, molecular docking, molecular dynamics (MD) and MM-PBSA calculations. J. Biomol. Struct. Dyn..

[41] Elmezayen AD, Al-Obaidi A, Şahin AT, Yelekçi K (2020). Drug repurposing for coronavirus (COVID-19): In silico screening of known drugs against coronavirus 3CL hydrolase and protease enzymes. J. Biomol. Struct. Dyn..

[42] Cava C, Bertoli G, Castiglioni I (2020). In silico discovery of candidate drugs against covid-19. Viruses.

[43] Wang J (2020). Fast identification of possible drug treatment of coronavirus disease-19 (COVID-19) through computational drug repurposing study. J. Chem. Inf. Model..

[44] Zhang D-H, Wu K-L, Zhang X, Deng S-Q, Peng B (2020). In silico screening of Chinese herbal medicines with the potential to directly inhibit 2019 novel coronavirus. J. Integr. Med..

[45] Liang J (2020). Interaction of the prototypical α-ketoamide inhibitor with the SARS-CoV-2 main protease active site in silico: Molecular dynamic simulations highlight the stability of the ligand-protein complex. Comput. Biol. Chem..

[46] Rawlings ND, Barrett AJ, Bateman A (2010). MEROPS: The peptidase database. Nucleic Acids Res.

[47] Chang MW, Ayeni C, Breuer S, Torbett BE (2010). Virtual screening for HIV protease inhibitors: A comparison of AutoDock 4 and Vina. PLoS ONE.

[48] Kumari R, Kumar R, Consortium D, Lynn A (2014). g_mmpbsa A GROMACS tool for high-throughput MM-PBSA calculations. J. Chem. Inf. Model..

[49] Zhang L (2020). Crystal structure of SARS-CoV-2 main protease provides a basis for design of improved α-ketoamide inhibitors. Science.

[50] Pearlman RS (1987). Rapid generation of high quality approximate 3D molecular structures. Chem. Des. Auto. News.

[51] Howser GB (2020). Computer Networks and the Internet: A Hands-on Approach.

[52] Spitzer R, Jain AN (2012). Surflex-Dock: Docking benchmarks and real-world application. J. Comput. Aided Mol..

[53] Trott O, Olson AJ (2010). AutoDock Vina: Improving the speed and accuracy of docking with a new scoring function, efficient optimization, and multithreading. J. Comput. Chem..

[54] Abraham MJ (2015). GROMACS: High performance molecular simulations through multi-level parallelism from laptops to supercomputers. SoftwareX.

[55] Dierynck I (2011). TMC310911, a novel human immunodeficiency virus type 1 protease inhibitor, shows in vitro an improved resistance profile and higher genetic barrier to resistance compared with current protease inhibitors. Antimicrob. Agents Chemother..

[56] Jensen PB (1991). Antagonistic effect of Aclarubicin on daunorubicin-induced cytotoxicity in human small cell lung cancer cells: relationship to DNA integrity and topoisomerase II. Cancer Res..

[57] Mimoto, T. *et al.* KNI-764, A novel dipeptide-based HIV protease inhibitor containing allophenylnorstatine. In *Peptide Science—Present and Future* 652–653 10.1007/0-306-46864-6_220 (1999).

[58] Galpin I, Wilby A, Place G, Beynon R (1984). Synthetic analogues of the proteinase inhibitor: Chymostatin. Int. J. Pept. Protein Res..

[59] Xue X (2008). Structures of two coronavirus main proteases: Implications for substrate binding and antiviral drug design. J. Med. Virol.

[60] Pires DE, Blundell TL, Ascher DB (2015). pkCSM: Predicting small-molecule pharmacokinetic and toxicity properties using graph-based signatures. J. Med. Chem..

[61] Irwin JJ, Shoichet BK (2005). ZINC—A free database of commercially available compounds for virtual screening. J Chem. Inf. Model..

[62] Noble S, Faulds D (1996). Saquinavir: A review of its pharmacology and clinical potential in the management of HIV infection. Drugs.

[63] Hall DC, Ji H-F (2020). A search for medications to treat COVID-19 via in silico molecular docking models of the SARS-CoV-2 spike glycoprotein and 3CL protease. Travel Med. Infect. Disease.

[64] Geronikaki A, Eleftheriou P, Poroikov V (2016). Anti-HIV Agents: Current status and recent trends. Commun. Dis. Dev. World.

[65] Zeuzem S (2013). Faldaprevir and deleobuvir for HCV genotype 1 infection. N. Engl. J. Med..

[66] Huey, R. & Morris, G.M. Using AutoDock 4 with AutoDocktools: A tutorial. In *The Scripps Research Institute, USA*, 54–56 (2008).

[67] Wang J, Wolf RM, Caldwell JW, Kollman PA, Case DA (2004). Development and testing of a general amber force field. J. Comput. Chem..

[68] Da Silva AWS, Vranken WF (2012). ACPYPE-Antechamber python parser interface. BMC Res. Notes.

[69] Levy RM, Zhang LY, Gallicchio E, Felts AK (2003). On the nonpolar hydration free energy of proteins: Surface area and continuum solvent models for the solute–solvent interaction energy. J. Am. Chem. Soc..

