# Supplementary material for: Rational approach toward COVID-19 main protease inhibitors via molecular docking, molecular dynamics simulation and free energy calculation
Source: Sci Rep. 2020 Oct 19;10:17716. doi: 10.1038/s41598-020-74468-0 (PMC7572583; doi:10.1038/s41598-020-74468-0)

Rational Approach toward COVID-19 Main Protease Inhibitors via Molecular Docking, Molecular Dynamics Simulation and Free Energy Calculation.

Seketoulie Keretsu[a], Swapnil P. Bhujbal[a], Seung Joo Cho[a,b,*]

[a] *Department of Biomedical Sciences, College of Medicine, Chosun University, Gwangju 501-759, Republic of Korea*

[b] *Department of Cellular Molecular Medicine, College of Medicine, Chosun University, Gwangju 501-759, Republic of Korea*

Supplementary Material

Table S1. The PubChem ID of the 100 compounds selected based on the total binding score from surflex dock and binding energy score from Autodock vina.

Sl. No	PubChem ID (CID)	Surflex Dock (Binding Score)	Autodock Vina (Binding Energy)	Sl. No	PubChem ID (CID)	Surflex Dock (Binding Score)	Autodock Vina (Binding Energy)
1	**441243**	8.6	-9.1	51	**25167947**	8.7	-7.9
2	**102207029**	7.0	-8.9	52	**52319945**	8.7	-7.9
3	**134691740**	7.0	-8.9	53	**5362440**	7.9	-7.9
4	**446837**	7.9	-8.8	54	**101248820**	7.8	-7.9
5	**11962092**	8.3	-8.7	55	**134712**	7.7	-7.9
6	**447216**	7.1	-8.7	56	**6918376**	7.7	-7.9
7	**134823859**	7.0	-8.7	57	**6440352**	7.3	-7.9
8	**451415**	7.0	-8.7	58	**86575820**	7.3	-7.9
9	**74353448**	7.7	-8.6	59	**11464980**	7.3	-7.9
10	**132585244**	7.2	-8.6	60	**5275815**	7.1	-7.9
11	**134815261**	7.1	-8.6	61	**86223086**	7.0	-7.9
12	**21881944**	7.0	-8.6	62	**16122592**	6.9	-7.9
13	**5492607**	10.5	-8.5	63	**102285029**	10.3	-7.8
14	**53361968**	7.1	-8.5	64	**132531950**	9.0	-7.8
15	**446918**	7.0	-8.5	65	**16126898**	8.3	-7.8
16	**92727**	9.7	-8.4	66	**445114**	8.3	-7.8
17	**100997107**	8.4	-8.4	67	**2977478**	8.0	-7.8
18	**15942730**	10.9	-8.3	68	**44302888**	7.6	-7.8
19	**103535**	10.5	-8.3	69	**92135659**	7.5	-7.8
20	**4322**	9.9	-8.3	70	**392622**	7.3	-7.8
21	**91668478**	7.2	-8.3	71	**177992**	7.3	-7.8
22	**5380091**	7.2	-8.3	72	**42641863**	7.2	-7.8
23	**9828551**	7.1	-8.3	73	**11273632**	9.1	-7.7

24	**185617**	7.1	-8.3	74	**11505008**	8.7	-7.7
25	**3451**	7.0	-8.3	75	**71481119**	8.4	-7.7
26	**20054919**	7.0	-8.3	76	**15221770**	8.4	-7.7
27	**70639872**	6.9	-8.3	77	**69496820**	8.2	-7.7
28	**6451084**	10.0	-8.2	78	**138110650**	8.1	-7.7
29	**6449855**	9.5	-8.2	79	**9547976**	8.0	-7.7
30	**5464035**	9.3	-8.2	80	**49862506**	7.4	-7.7
31	**129894295**	9.1	-8.2	81	**65016**	7.2	-7.7
32	**44425681**	8.3	-8.2	82	**11249248**	7.2	-7.7
33	**6918046**	10.7	-8.1	83	**16760337**	8.9	-7.6
34	**45358152**	9.9	-8.1	84	**66577056**	7.9	-7.6
35	**46178275**	9.2	-8.1	85	**49862502**	7.6	-7.6
36	**443119**	9.1	-8.1	86	**3035562**	7.5	-7.6
37	**71588937**	7.1	-8.1	87	**10462207**	7.2	-7.6
38	**46936189**	7.1	-8.1	88	**3081200**	6.9	-7.6
39	**65023**	9.6	-8	89	**46850523**	9.7	-7.5
40	**6324659**	9.3	-8	90	**49771527**	9.1	-7.5
41	**5311140**	9.2	-8	91	**60927**	9.1	-7.5
42	**3849**	8.4	-8	92	**350804**	8.9	-7.5
43	**91801117**	7.5	-8	93	**506900**	8.7	-7.5
44	**213039**	7.5	-8	94	**16720766**	8.0	-7.5
45	**5492587**	7.4	-8	95	**5487540**	7.9	-7.5
46	**449247**	7.4	-8	96	**75641285**	7.5	-7.5
47	**6918784**	7.3	-8	97	**10458621**	7.4	-7.5
48	**53362096**	7.1	-8	98	**4339**	7.2	-7.5
49	**154072**	7.1	-8	99	**11733963**	7.0	-7.5
50	**5748613**	7.0	-8	100	**11466507**	9.1	-7.4

Table S2: The predicted ADMET values for the 10 selected compounds.

Compounds	Absorption	Distribution	Metabolism							Excretion	Toxicity
	Intestinal absorption (human)	VDss (human)	CYP							Total Clearance	AMES toxicity
			**2D6**	**3A4**	**1A2**	**2C19**	**2C9**	**2D6**	**3A4**		
			substrate		inhibitor						
	Numeric (% Absorbed)	Numeric (log L/kg)	Categorical (Yes/No)							Numeric (log ml/min/kg)	Categorical (Yes/No)
**441243**	57	1.0	No	Yes	No	No	No	No	Yes	0.3	No
**451415**	84	1.4	No	Yes	No	No	No	No	Yes	0.9	No
**446837**	63	0.7	No	Yes	No	Yes	Yes	No	Yes	-0.1	No
**53361968**	82	1.9	No	Yes	No	No	No	No	Yes	0.2	No
**46178275**	53	-0.6	No	Yes	No	No	No	No	No	0.3	No
**9828551**	66	0.25	No	Yes	No	No	No	No	Yes	0.5	No
**644196**	39	0.3	No	Yes	No	No	No	No	Yes	1.0	No
**134815261**	92	0.85	No	Yes	No	No	No	No	Yes	0.4	No
**15942730**	17	-1.3	No	No	No	No	No	No	Yes	0.03	No
**132531950**	75	0.5	No	Yes	No	No	No	No	No	0.5	No

Figure S1. Structure of the inhibitor **N3** in complex with 3CL^{pro} as given in **6LU7**. The protein and ligand were shown in gray and magenta color. The mesh (cyan) representation between domain 1 and domain 2 represents the area to be searched by surflex dock (protomol).

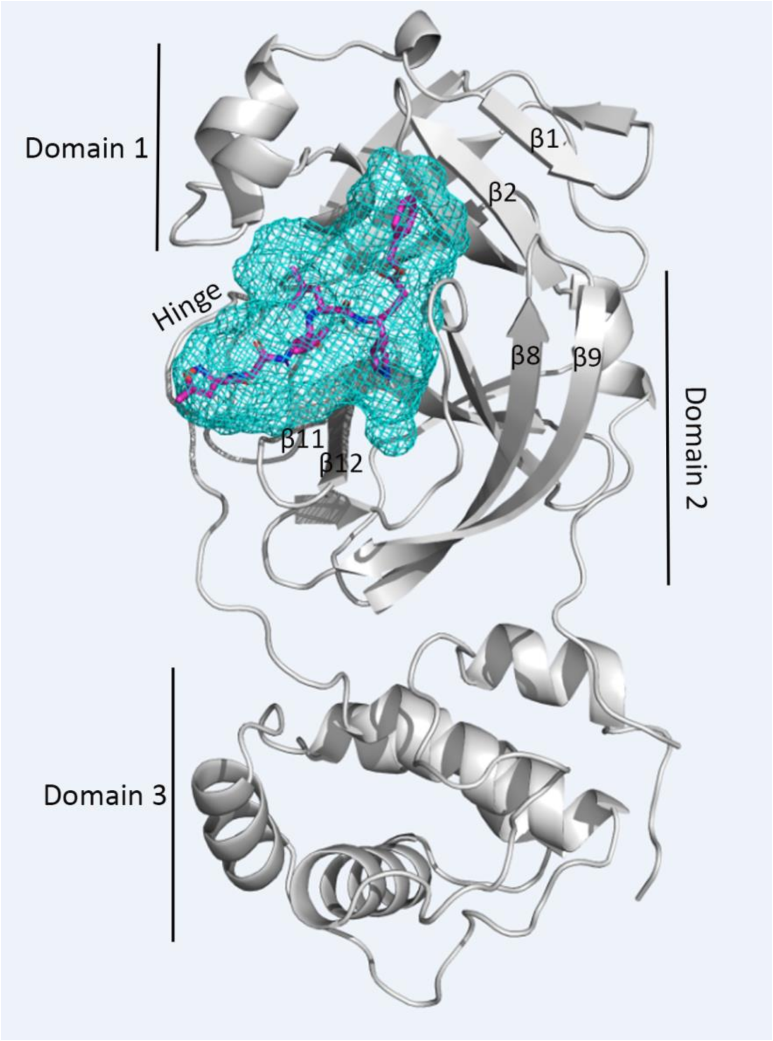

Figure S2. Showing the H-bond interactions of the inhibitors **N3** and **13b** with 3CL^{pro} from the molecular docking studies. H-bond interactions were represented by yellow dotted lines and residues forming H-bonds were shown in purple color. **(a)** Binding interactions between **N3** and 3CL^{pro}. **(b)** Binding interactions between **13b** and 3CL^{pro}.

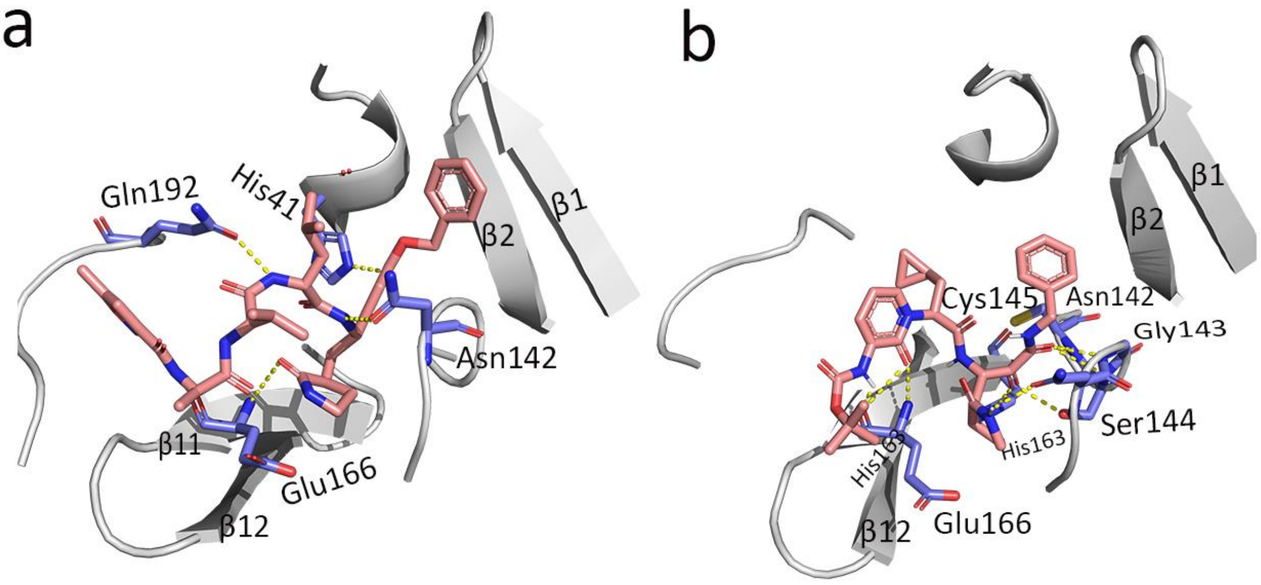

Figure S3. Showing the hydrophobic interactions of the inhibitors with 3CL^{pro}. Hydrophobic interactions were represented in red dotted lines. Residues that showed Hydrophobic and H-bond interactions were given in green and black label respectively. **(a) 451415**-3CL^{pro} **(b) 53361968**-3CL^{pro} **(c) 446837**-3CL^{pro} **(d) 446837**-3CL^{pro}.

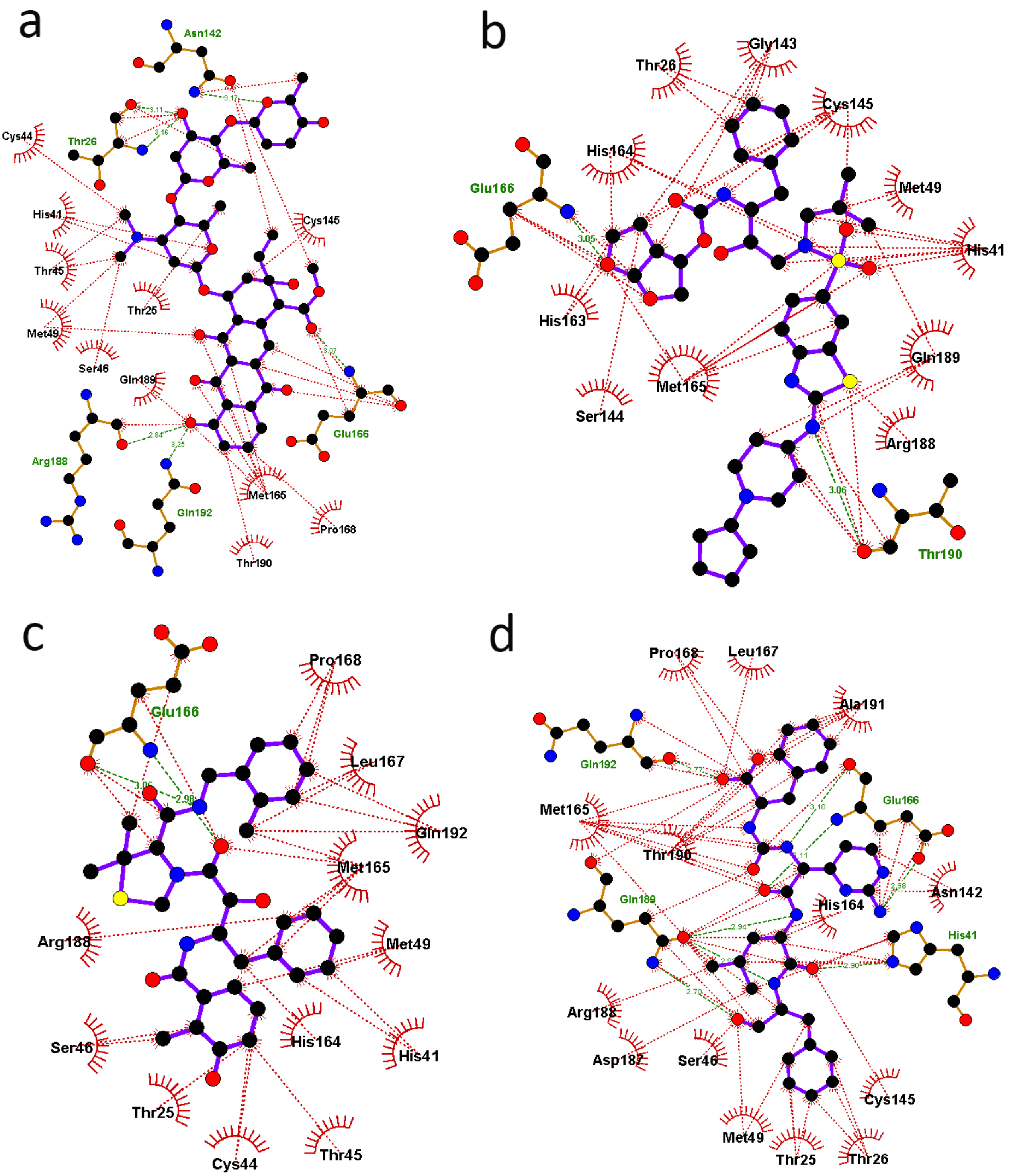

Figure S4. The binding pose of the compounds inside 3CL^{pro} at 47th ns (yellow), 48th ns (magenta), 49th ns (cyan), and 50th ns (green) of the MD simulation. **(a) N3**-3CL^{pro} **(b) 13b**-3CL^{pro} **(c) 451415**-3CL^{pro} **(d) 53361968**-3CL^{pro}.

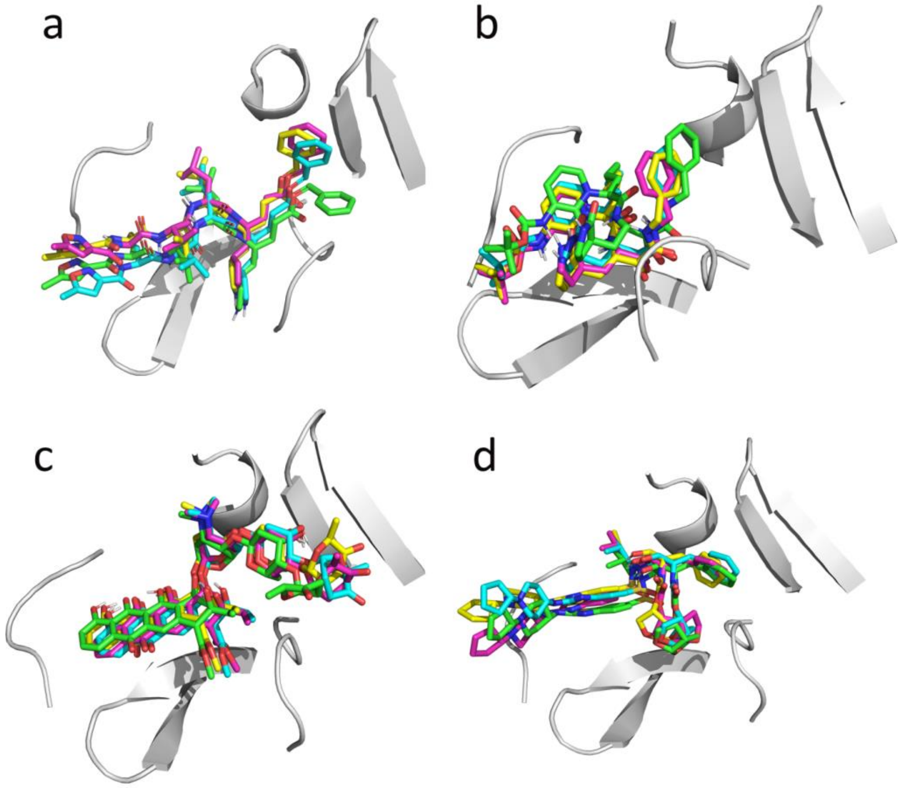

Figure S5. The overlap between the crystal ligand pose (green) and the docked pose (salmon) of **N3** and **13b** at the binding site of 3CL^{pro}.**(a) N3** inside 3CL^{pro} **(b) 13b** inside 3CL^{pro}

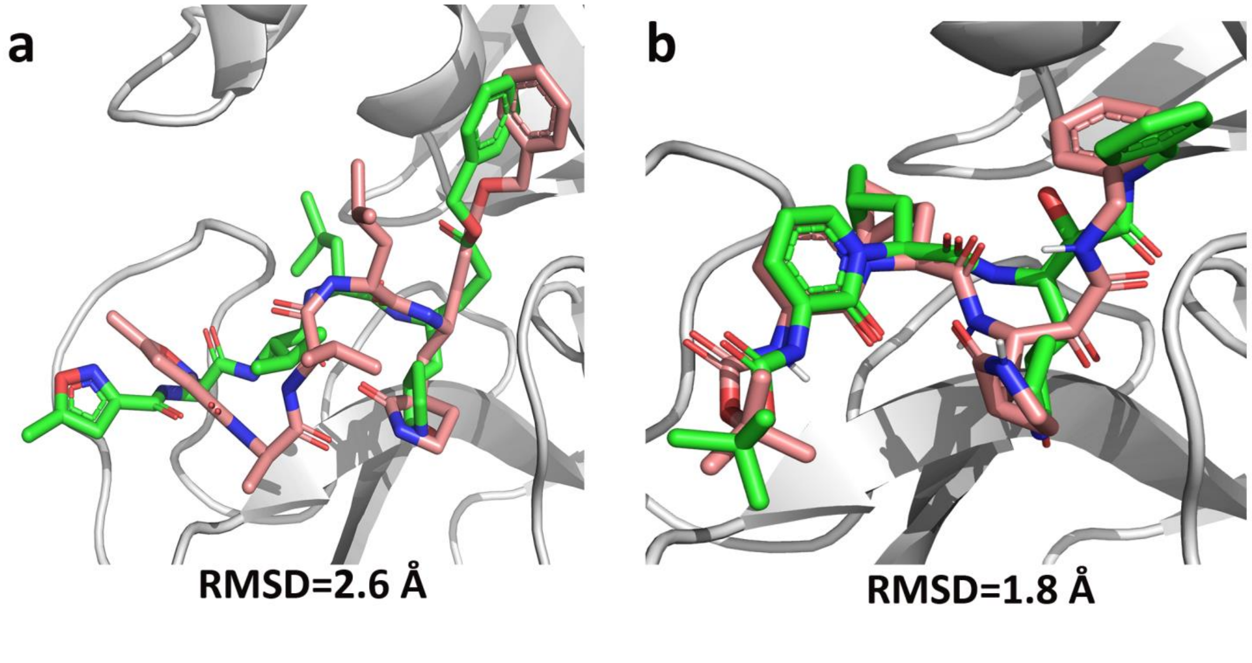

Supplement: Supplementary file 1 — Supplementary Information 1 [file 41598_2020_74468_MOESM1_ESM.pdf]
